# Advances in Breast Cancer Diagnostics: From Screening to Precision Medicine

**DOI:** 10.3390/diagnostics16081181

**Published:** 2026-04-16

**Authors:** Klaudia Kubiak, Joanna Bidzińska, Marta Bednarek, Edyta Szurowska

**Affiliations:** 1 Department of Pharmaceutical Pathophysiology, Medical University of Gdansk, 80-210 Gdańsk, Poland; 2 Second Department of Radiology, Medical University of Gdańsk, 80-210 Gdańsk, Poland; jbidzinska@gumed.edu.pl (J.B.); marta.bednarek@gumed.edu.pl (M.B.); edyta.szurowska@gumed.edu.pl (E.S.); 3 Department of Radiology, University Clinical Centre, 80-210 Gdańsk, Poland; 4 Central Laboratory, University Clinical Centre, 80-210 Gdańsk, Poland

**Keywords:** breast cancer, screening, breast imaging, artificial intelligence, liquid biopsy, circulating tumor DNA, genomic profiling, precision medicine, next-generation sequencing, molecular diagnostics

## Abstract

Breast cancer remains the most frequently diagnosed malignancy in women worldwide, accounting for approximately 2.3 million new cases and 670,000 deaths annually. The diagnostic landscape has undergone a paradigm shift over the past two decades, evolving from morphology-based classification toward molecularly informed, precision-guided strategies. Early and accurate diagnosis is fundamental to improving outcomes; advances in imaging technology, including digital breast tomosynthesis (DBT), contrast-enhanced mammography (CEM), and abbreviated magnetic resonance imaging (MRI), have improved sensitivity and specificity in diverse patient populations. Simultaneously, the integration of artificial intelligence (AI) and radiomics into screening workflows offers unprecedented potential for risk stratification and a reduction in false-positives. At the pathological level, multi-gene expression profiling assays such as Oncotype DX, MammaPrint, Prosigna, and EndoPredict have refined prognostic classification and guide adjuvant chemotherapy decisions in early-stage hormone receptor-positive disease. The emergence of liquid biopsy, circulating tumor DNA (ctDNA), circulating tumor cells (CTCs), and exosomal biomarkers provides minimally invasive tools for real-time monitoring of response, residual disease, and the evolution of resistance mechanisms. Precision diagnostics now encompass next-generation sequencing (NGS)-based comprehensive genomic profiling, enabling identification of actionable alterations such as PIK3CA mutations, HER2 amplification, BRCA1/2 pathogenic variants, and NTRK fusions, each linked to approved therapeutic agents. The purpose of this review is to provide a comprehensive synthesis of current and emerging diagnostic modalities in breast cancer—from population-level screening to individualized molecular profiling—and to examine how integrative, multimodal diagnostic platforms are reshaping clinical decision-making in the era of precision medicine.

## 1. Introduction

Breast cancer is a heterogeneous malignancy comprising distinct biological subtypes with markedly different natural histories, therapeutic vulnerabilities, and prognoses. According to estimates from the GLOBOCAN 2022 database (still the primary reference in 2026 analyses), breast cancer accounted for approximately 2.3 million new cases and 670,000 deaths in women worldwide annually [1]. Despite improvements in survival rates attributed to organized screening programs and systemic therapeutic advances, breast cancer remains the leading cause of cancer-related mortality in women in most low- and middle-income countries [2]. The five-year relative survival rate in the United States approaches 92% overall but remains approximately 33% for metastatic disease, underscoring the critical importance of early detection [3].

Historically, breast cancer diagnosis has relied on two foundational pillars: radiological imaging and histopathological evaluation. Over the past two decades, however, the diagnostic landscape has expanded dramatically. The genomic era has elucidated the molecular underpinnings of breast cancer, enabling its reclassification into intrinsic subtypes—Luminal A, Luminal B, Human Epidermal Growth Factor Receptor 2 (HER2)-enriched, and Basal-like/Triple-negative—that more accurately predict clinical behavior and therapeutic response than traditional immunohistochemical assessment of hormone receptors and HER2 alone [4,5]. Multi-gene expression panels have translated research-level molecular subtyping into clinically actionable prognostic and predictive instruments, sparing tens of thousands of women from unnecessary chemotherapy each year [6].

Concurrently, imaging technologies have undergone significant changes. Digital mammography is increasingly being complemented or replaced by digital breast tomosynthesis in high-income settings, improving cancer detection rates, particularly in women with dense breast tissue [7]. Emerging modalities such as contrast-enhanced mammography and abbreviated Magnetic Resonance Imaging (MRI) protocols offer cost-effective supplemental screening options for individuals at intermediate to high risk [8,9]. The integration of artificial intelligence and deep learning into mammographic and tomosynthesis interpretation represents a potentially transformative development. For example, McKinney et al. [10] demonstrated that an AI system showed superior performance to radiologists and, when participating in the UK double-reading workflow, reduced the workload of the second reader by 88%, while multiple other reader studies have confirmed non-inferiority or even superiority of AI systems compared with experienced radiologists in controlled environments.

Beyond tissue-based diagnostics, liquid biopsy technologies have introduced a fundamentally new dimension to breast cancer detection and monitoring. Analysis of circulating tumor DNA (ctDNA), circulating tumor cells (CTCs), and tumor-derived extracellular vesicles enable non-invasive genomic profiling, early detection of minimal residual disease, and real-time tracking of clonal evolution under therapeutic pressure [11,12]. In parallel, the approval of targeted therapies directed at CDK4/6, PI3Kα, HER2, and the PARP pathway has rendered companion diagnostic testing an indispensable component of treatment selection, particularly in advanced diseases [13].

The primary objective of this review is to provide a comprehensive, up-to-date synthesis of the rapidly evolving diagnostic landscape in breast cancer, spanning the full continuum from population-level screening to advanced molecular profiling and real-time monitoring with liquid biopsy. Specifically, we aim to integrate advances across imaging modalities, artificial intelligence applications, histopathological and immunohistochemical biomarkers, multi-gene expression assays, comprehensive genomic profiling, and emerging liquid biopsy technologies while examining their synergistic role in enabling precision medicine. Although several excellent reviews have addressed individual components of breast cancer diagnostics (e.g., imaging or genomic testing), there remains a need for an integrative overview that emphasizes technological convergence, multimodal workflows, implementation challenges across diverse healthcare settings, and the translation of diagnostic innovations into improved clinical decision-making. By consolidating evidence from landmark trials, recent meta-analyses, and emerging 2023–2026 data, this review fills that gap and offers clinicians and researchers a unified framework for understanding how contemporary diagnostic platforms are reshaping risk stratification, early detection, treatment selection, and disease monitoring in breast cancer.

This review was conducted following established principles for narrative and scoping reviews in oncology. A comprehensive literature search was performed in the electronic databases PubMed, Embase, Scopus, and Web of Science, supplemented by targeted searches of major clinical guideline repositories (NCCN, ASCO, ESMO, ACR) and ClinicalTrials.gov. The search covered the period from January 2015 to January 2026, while foundational studies published before 2015 were included when they represented landmark trials or seminal contributions. Search terms combined keywords and controlled vocabulary (MeSH/Emtree) related to “breast cancer,” “diagnosis,” “screening,” “imaging,” “artificial intelligence,” “liquid biopsy,” “circulating tumor DNA,” “genomic profiling,” “next-generation sequencing,” and “precision medicine.” Inclusion criteria encompassed peer-reviewed original research articles, meta-analyses, systematic reviews, large prospective/retrospective cohort studies, and randomized controlled trials with direct relevance to breast cancer diagnostics. Exclusion criteria included case reports, small case series editorials, and studies lacking sufficient methodological detail or validation.

**Figure 1:**
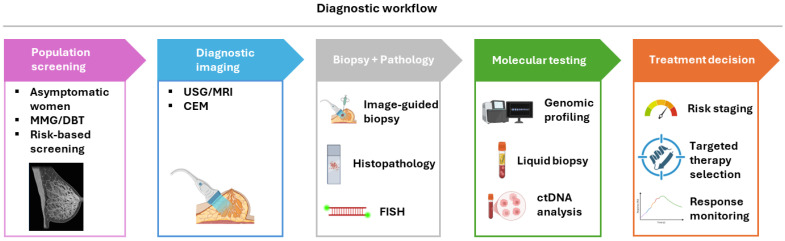
Stepwise diagnostic and decision-making workflow in breast cancer. The figure illustrates the contemporary multimodal diagnostic pathway, progressing from population-level screening in asymptomatic individuals to diagnostic imaging in patients with abnormal findings or clinical symptoms. Suspicious lesions undergo image-guided biopsy followed by histopathological evaluation and immunohistochemical biomarker assessment. Subsequent molecular testing, including gene expression profiling and genomic analysis, refines risk stratification and identifies actionable targets. These integrated data inform personalized treatment decision-making, including therapy selection and disease monitoring.

Figure 1 provides an overview of the contemporary multimodal diagnostic workflow in breast cancer.

## 2. Imaging-Based Screening Modalities

The following sections review the principal imaging modalities deployed across the breast cancer diagnostic continuum, from established population-level screening tools to supplemental and emerging technologies, organized broadly from most to least widely implemented. For each modality, clinical performance data, guideline status, and practical limitations are considered. A comparative summary of imaging modalities, including their advantages and limitations, is provided in Table 1.

### 2.1. Conventional Digital Mammography

Screen-film mammography, introduced in the 1960s, provided the evidence-based foundation for organized population screening. Eight randomized controlled trials conducted between 1963 and 1991 demonstrated a 15–30% relative reduction in breast cancer mortality among invited women aged 40–74 years, leading to the establishment of national screening programs across Europe and North America [14,15]. The transition to full-field digital mammography (FFDM) in the late 1990s and 2000s improved image quality, enabled computer-aided detection (CAD), and facilitated teleradiology and archiving. The Digital Mammographic Imaging Screening Trial (DMIST) demonstrated FFDM superiority over film in premenopausal women and those with dense breasts [16].

Nonetheless, conventional 2D mammography retains fundamental limitations: tissue superimposition can obscure lesions (false negatives) or mimic pathology (false positives). Sensitivity ranges from 77 to 95% in fatty breasts but declines to 30–48% in extremely dense breasts [17]. Screening generates recall rates of 10–13% in the United States, with positive predictive value for biopsy of 25–40% [18]. Despite its limitations, mammography remains the only modality with proven mortality reduction in randomized clinical trials.

### 2.2. Digital Breast Tomosynthesis

Digital breast tomosynthesis (DBT) acquires multiple low-dose X-ray projections and reconstructs 1 mm slices, reducing superimposition [19,20]. Prospective and retrospective studies, including recent meta-analyses, show that DBT + FFDM increases cancer detection by 1–2.7 per 1000 women screened versus FFDM alone, with recall reductions of 15–40% [21,22]. The Oslo Tomosynthesis Screening Trial reported a 40% increase in invasive cancer detection and 15% reduction in false positives with DBT + synthetic 2D versus FFDM [23]. The STORM-2 trial confirmed superiority in European populations [24].

Evidence indicates that DBT preferentially detects invasive, node-negative cancers, mitigating overdiagnosis concerns [25]. Synthetic 2D reconstruction limits dose to ~20–30% above FFDM, within safety thresholds [26]. Recent data support DBT benefits in family history cohorts and dense breasts. However, increased radiation exposure and higher implementation costs remain important considerations for widespread adoption.

### 2.3. Contrast-Enhanced Mammography

Contrast-enhanced mammography (CEM) highlights neoangiogenesis compared with dual-energy subtraction post-iodinated contrast. Studies report sensitivity of 93–100%, outperforming mammography and rivaling abbreviated MRI in high-risk/dense populations [27,28]. The CREST trial and others position CEM as cost-effective for intermediate-risk supplemental screening, especially in dense breasts where MRI access is limited [29].

Limitations include contrast contraindications, radiation, and slightly lower specificity than MRI. Emerging data support preoperative staging [30]. Ongoing trials (CMIST, BRAID interim results) show high CDR (~19/1000) comparable to abbreviated MRI in dense breasts post-negative mammogram. However, the need for iodinated contrast limits its use in patients with renal impairment or contrast allergy.

### 2.4. Breast Magnetic Resonance Imaging

Dynamic contrast-enhanced breast MRI (DCE-MRI) is established as the most sensitive modality for breast cancer detection, with reported sensitivity of 77–100% across a range of protocols and patient populations—the lower end reflecting abbreviated or earlier-generation protocols, the upper end observed in high-risk surveillance cohorts on full DCE sequences [31]. Beyond detection, DCE-MRI characterizes ipsilateral disease extent, identifies occult contralateral malignancy, and evaluates chest wall involvement. ACR Appropriateness Criteria and NCCN guidelines recommend annual supplemental MRI for women with a lifetime breast cancer risk ≥ 20%, confirmed BRCA1/2 mutation carriers, or those with a history of thoracic irradiation between ages 10 and 30 years [32].

Resource intensity remains the principal barrier to widespread adoption: standard DCE-MRI protocols require 25–45 min of scanner time, dedicated equipment, subspecialty-trained radiologists, and contrast administration [8,33]. Abbreviated MRI (AB-MRI) protocols requiring as little as 3–10 min have demonstrated diagnostic performance comparable to full protocols for screening purposes across multiple prospective studies, including the EA1141 trial, which reported cancer detection rates equivalent to standard MRI [8]. Ultrafast DCE-MRI sequences that capture first-pass contrast bolus kinetics further improve lesion characterization by assessing peak enhancement dynamics.

The DENSE trial demonstrated a significant reduction in interval cancers with supplemental MRI screening in women with extremely dense breasts. Follow-up analyses and meta-analyses have confirmed a sustained reduction of approximately 50% in interval cancer rates. Ongoing trials, such as the MyPeBS study, continue to evaluate risk-adapted screening strategies incorporating MRI [34,35,36].

Diffusion-weighted imaging (DWI) as an adjunct or potential contrast-free alternative to DCE-MRI is under active investigation. Apparent diffusion coefficient (ADC) mapping provides quantitative tissue characterization that correlates with histological grade and receptor status and may reduce reliance on gadolinium-based contrast in future abbreviated protocols [37].

### 2.5. Breast Ultrasound

Breast ultrasound (USG) is widely used as an adjunct to mammography for characterization of palpable and mammographically detected lesions, evaluation of dense breast parenchyma, and guidance of interventional procedures. Handheld ultrasound has a longstanding role but is highly operator-dependent and time-consuming for whole-breast screening applications. Automated breast ultrasound (ABUS) systems acquire standardized volumetric images of the entire breast with coronal reconstruction, reducing operator variability and enabling remote reading [38].

Meta-analyses of supplemental ABUS screening in dense-breast populations report additional cancer detection of 2.0–4.6 per 1000 women, at the cost of substantially elevated recall rates (13–19%) and biopsy rates [39]. Point-of-care ultrasound using handheld devices is increasingly employed in low-resource settings as a primary screening tool, with reasonable performance when delivered by trained practitioners. Elastography techniques, shear-wave and strain elastography, improve specificity by assessing lesion stiffness, a surrogate for desmoplastic stromal reaction, reducing unnecessary biopsies for probably benign lesions [40]. Despite its advantages, ultrasound is associated with high false-positive rates and limited reproducibility in screening settings.

### 2.6. Emerging and Supplemental Technologies

Molecular breast imaging (MBI), utilizing technetium-99 m sestamibi and dedicated cadmium zinc telluride gamma cameras, detects tumors based on differential radiotracer uptake reflecting mitochondrial density and tissue perfusion. MBI has demonstrated sensitivity of 91% in dense breasts compared with 25% for mammography in the same population, at acceptable radiation doses (approximately 4.3–6.0 mGy effective dose) [41]. Positron emission mammography (PEM) with fluorine-18-fluorodeoxyglucose (18F-FDG) offers extremely high spatial resolution (approximately 1.6 mm) for dedicated breast imaging, with reported sensitivity of 85–91% [42].

Electrical impedance tomography, thermography, and optical coherence tomography remain predominantly investigational, lacking robust clinical validation in unselected screening populations. Photoacoustic imaging, combining optical contrast with acoustic resolution, offers the promise of functional and molecular imaging without ionizing radiation or contrast agents, with several research groups reporting promising early-phase results [43].

Overall, no single imaging modality provides optimal sensitivity and specificity across all patient populations. While MRI demonstrates the highest sensitivity, its lower specificity and limited accessibility restrict its use as a universal screening tool. Conversely, mammography remains the cornerstone of population screening due to its availability and cost-effectiveness, despite reduced sensitivity in dense breast tissue. Emerging modalities such as DBT and CEM aim to bridge this gap by improving detection rates while maintaining feasibility in broader clinical settings.

## 3. Artificial Intelligence in Breast Cancer Imaging

Artificial intelligence (AI) is rapidly transforming breast cancer imaging, shifting diagnostics from purely visual interpretation toward data-driven, quantitative decision support systems.

### 3.1. Deep Learning for Image Interpretation

The application of convolutional neural networks (CNNs) and deep learning architectures to mammographic image analysis has rapidly advanced in recent years. Large-scale studies have demonstrated that AI systems trained on high-quality annotated datasets can achieve diagnostic performance comparable to, or exceeding, that of experienced radiologists. Foundational work by Rajpurkar et al. [44] and McKinney et al. [10] demonstrated that AI systems trained on large datasets could match or exceed individual radiologist performance from mammograms. The latter study, utilizing a dataset of 76,000 UK National Health Service screening mammograms, showed that AI reduced false positives by 5.7% and false negatives by 9.4%, with generalizability confirmed across a US dataset.

The prospective ScreenTrustCEM trial and the randomized Transpara AI-assisted reading trial have added clinical validity, demonstrating that AI-assisted double reading is non-inferior to conventional double reading while substantially reducing radiologist workload [45]. AI systems have demonstrated utility in the detection of subtle lesions—microcalcification clusters, architectural distortions, and asymmetries—which represent a disproportionate share of interval cancers and are prone to perceptual misses [46]. AI-generated malignancy scores at the level of individual lesions also show promise for risk stratification, enabling personalized screening intervals. However, most studies have been conducted in controlled or retrospective settings, and real-world performance may be influenced by variability in imaging quality, patient populations, and workflow integration.

Beyond traditional image interpretation on mammography and tomosynthesis, artificial intelligence is increasingly applied across the broader diagnostic pathway. In digital pathology, deep learning models applied to hematoxylin and eosin (H&E)-stained whole-slide images have demonstrated strong performance in automated tumor subtyping, histological grading, and biomarker quantification, including estrogen receptor, progesterone receptor, HER2, and Ki-67 status, often achieving concordance with expert pathologists [47,48]. These tools also show promise for predicting molecular subtypes directly from routine histology slides, potentially reducing reliance on additional immunohistochemical testing in resource-constrained settings.

In the domain of radiogenomics and mutation prediction, multimodal AI models integrating radiomic features from mammography or ultrasound with clinical data can non-invasively predict key actionable alterations such as PIK3CA mutations, offering a complementary approach to tissue-based NGS [49]. Furthermore, AI-enhanced risk assessment tools that combine mammographic features with polygenic risk scores or longitudinal imaging data are improving short- and intermediate-term risk stratification beyond traditional models, supporting more personalized screening intervals [50].

**Figure 2:**
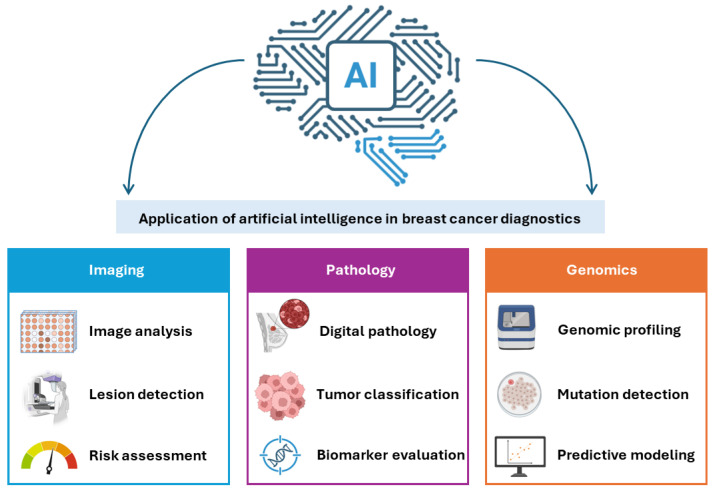
Artificial intelligence applications across the breast cancer diagnostic pathway. This figure illustrates the role of artificial intelligence (AI) across key domains of breast cancer diagnostics, including imaging, pathology, and genomics. In imaging, AI supports lesion detection, classification, and risk assessment across modalities such as mammography, ultrasound, and MRI. In pathology, AI enables automated analysis of digitized histological slides, including tumor detection, grading, and prediction of biomarker status and molecular subtypes. In genomics, AI facilitates the integration and interpretation of high-dimensional molecular data, supporting mutation prediction, risk stratification, and treatment selection.

These applications, summarized in Figure 2, illustrate the expanding role of AI from perceptual tasks in radiology toward integrative, multimodal decision support that spans imaging, pathology, genomics, and risk prediction.

### 3.2. Radiomics and Imaging Biomarkers

Radiomics is an emerging field that enables high-throughput extraction of quantitative imaging features—such as shape, texture, and intensity—allowing for the characterization of tumor heterogeneity beyond visual assessment. Applied to mammography, tomosynthesis, ultrasound, and MRI, radiomic signatures have demonstrated associations with histological grade, molecular subtype, lymph node status, pathological complete response to neoadjuvant chemotherapy, and recurrence risk [51,52].

Imaging genomics (radiogenomics) studies correlating radiomic features with genomic alterations or gene expression profiles have identified imaging surrogates for BRCA1/2 mutation status, PIK3CA mutation, immune microenvironment composition, and proliferation indices. While these findings are biologically informative and potentially clinically useful for guiding biopsy and treatment planning, radiomic models are highly sensitive to acquisition parameters, segmentation methods, and feature extraction software, necessitating rigorous standardization efforts such as those promoted by the Image Biomarker Standardization Initiative (IBSI) before widespread clinical translation [53]. Despite promising results, lack of standardization and reproducibility remain a major barrier to clinical implementation. Integration of radiomics with AI-based models may further enhance predictive accuracy and enable the development of robust, clinically applicable decision-support systems.

### 3.3. Implementation Challenges and Regulatory Landscape

Despite demonstrated analytical performance, integration of AI into real-world clinical screening programs faces substantial challenges. These include algorithmic bias arising from underrepresentation of diverse racial and ethnic groups in training datasets, with documented performance disparities for Black women and women with dense breasts [54].

Recent evidence highlights effective mitigation strategies through federated learning applied to multi-ethnic and multi-vendor datasets. These approaches enable collaborative model training across institutions without sharing raw patient data, significantly improving fairness, generalizability, and equity while maintaining privacy (via differential privacy techniques achieving >96% accuracy in diverse cohorts) [55].

Regulatory approval pathways vary by jurisdiction: the FDA has granted 510(k) clearance to multiple AI CAD devices but distinguishes between software functioning as a physician substitute versus an adjunct. Explainability, the ability of AI systems to provide interpretable and transparent decision-making processes, remains limited in many deep learning models and is a requisite for radiologist trust and medicolegal acceptance [56]. Prospective integration studies and health technology assessments are required to establish cost-effectiveness and patient-level outcome benefits at the population level.

While these advances hold substantial promise, successful real-world implementation faces amplified challenges in low-resource and heterogeneous healthcare settings. Algorithmic bias remains a critical issue when models are predominantly trained on data from high-income countries, leading to reduced performance in diverse ethnic groups, dense-breast populations, or underrepresented regions [57]. In low- and middle-income countries, additional barriers include limited digital pathology infrastructure, unreliable internet connectivity for cloud-based AI, high costs of computational resources, and scarcity of locally validated datasets, all of which can widen existing disparities in breast cancer outcomes [58,59]. Federated learning and lightweight, task-oriented AI models offer potential mitigation strategies by enabling collaborative training without raw data sharing and reducing hardware demands. Prospective, multi-center validation studies that deliberately include low-resource cohorts, combined with explainable AI techniques to build clinician trust, will be essential to ensure equitable translation of these technologies across global healthcare systems.

Collectively, while AI demonstrates substantial potential to enhance breast cancer detection and workflow efficiency, its successful clinical integration will depend on robust validation, standardization, and alignment with regulatory and ethical frameworks.

## 4. Pathological Diagnosis and Tissue Biomarkers

Pathological evaluation and biomarker assessment remain central to breast cancer diagnosis, enabling definitive classification and guiding personalized therapeutic strategies.

### 4.1. Image-Guided Biopsy Techniques

Histopathological tissue sampling remains the gold standard for breast cancer diagnosis, providing definitive morphological characterization, biomarker assessment, and the foundation for treatment planning. Image-guided percutaneous core needle biopsy (CNB) has supplanted surgical excision biopsy as the preferred diagnostic modality, offering equivalent diagnostic accuracy with lower morbidity, cost, and time to diagnosis [60]. Vacuum-assisted biopsy (VAB) devices retrieve multiple larger-core specimens through a single insertion, improving sampling adequacy for microcalcifications and small lesions.

Stereotactic biopsy targets microcalcifications detectable only mammographically, while ultrasound-guided biopsy is preferred for masses and lymph nodes [61,62]. MRI-guided vacuum-assisted biopsy addresses lesions identified only on MRI, so-called MRI-only lesions, that are not amenable to conventional guidance. Concordance rates between MRI-guided biopsy and surgical pathology approach 94–97%, with discordance necessitating repeat sampling or excision [63]. However, sampling error and tumor heterogeneity remain potential limitations, particularly in small or heterogeneous lesions.

### 4.2. Histopathological Classification

The WHO Classification of Tumors of the Breast (5th edition, 2019) recognizes over 20 invasive breast cancer histological subtypes, with invasive carcinoma of no special type (NST, formerly invasive ductal carcinoma) constituting approximately 70–80% of cases [64]. Special-type carcinomas, including lobular (10–15%), tubular, mucinous, medullary-like, adenoid cystic, and metaplastic subtypes, carry distinct prognostic and therapeutic implications. Grading by the modified Bloom-Richardson-Elston (Nottingham) system (assessing tubule formation, nuclear pleomorphism, and mitotic rate) provides independent prognostic information complementary to molecular subtyping [65]. Despite detailed histopathological classification, interobserver variability and overlap between subtypes may limit reproducibility in routine clinical practice.

Ductal carcinoma in situ (DCIS) represents a non-obligate precursor to invasive cancer; its diagnosis, increasingly common in the mammographic screening era, poses significant management challenges due to heterogeneous biological risk and lack of consensus regarding optimal treatment for low-grade lesions. The COMET and LORD trials are actively investigating active surveillance as an alternative to immediate treatment for low-risk DCIS [66]. This has raised concerns regarding overdiagnosis and overtreatment, particularly in low-grade lesions detected through screening programs.

### 4.3. Biomarker Assessment by Immunohistochemistry

Accurate biomarker assessment is critical, as it directly determines systemic treatment selection and patient outcomes. Estrogen receptor (ER), progesterone receptor (PR), and HER2 immunohistochemistry constitute the mandatory biomarker panel for all newly diagnosed invasive breast cancers, defining the major therapeutic categories: hormone receptor-positive/HER2-negative (HR+/HER2−, approximately 70%), HER2-positive (HER2+, approximately 15–20%), and triple-negative (TNBC, approximately 15%) [67] (Table 2). The 2023 ASCO/CAP guidelines define ER positivity as ≥1% nuclear staining but recommend reporting of results as ER-low-positive (1–10%) versus ER-positive (≥10%), given emerging evidence that ER-low tumors exhibit biological and therapeutic behavior resembling TNBC [68]. HER2 scoring by IHC (0, 1+, 2+, 3+) with reflex in situ hybridization (ISH) for 2+ cases has been refined to incorporate HER2-ultralow (IHC 0 with faint incomplete staining in ≥10% of cells) and HER2-low (IHC 1+ or IHC 2+/ISH-negative) categories, following regulatory approval of trastuzumab deruxtecan (T-DXd) for HER2-low metastatic breast cancer [69].

The Ki-67 proliferation index, assessed by MIB-1 antibody staining, provides complementary prognostic information but suffers from substantial inter-laboratory variability, limiting its standalone clinical utility. Standardization initiatives, including the International Ki-67 in Breast Cancer Working Group phase 3 ring study, have defined analytical and preanalytical requirements for reproducible Ki-67 reporting [70]. As a result, Ki-67 is primarily used as a complementary marker rather than a definitive decision-making tool. Tumor-infiltrating lymphocytes (TILs) assessed on hematoxylin and eosin sections are now recognized as a standardized prognostic and predictive biomarker, particularly in TNBC and HER2+ subtypes, with stromal TIL counts predicting pathological complete response to neoadjuvant chemotherapy and correlating with event-free survival [71]. Standardization of TIL assessment may further enhance its role in guiding immunotherapy decisions.

### 4.4. Circulating Tumor Markers

Circulating tumor markers have historically been investigated as minimally invasive tools for breast cancer detection and monitoring. The most evaluated serum biomarkers include cancer antigen 15-3 (CA 15-3), cancer antigen 27.29 (CA 27.29), and carcinoembryonic antigen (CEA). These markers are derived from tumor-associated glycoproteins shed into the bloodstream and can be measured using standard immunoassays.

Despite their widespread availability, circulating tumor markers have limited sensitivity and specificity for early-stage breast cancer and are therefore not recommended for population screening or primary diagnosis [72,73]. Elevated levels may also be observed in benign conditions, including liver disease and inflammatory states, further limiting their diagnostic accuracy.

Current clinical guidelines, including those from ASCO and ESMO, do not recommend the routine use of CA 15-3, CA 27.29, or CEA for screening or diagnosis but acknowledge their potential role as adjunctive tools in treatment monitoring and follow-up in selected patients with metastatic disease [72,73,74]. Overall, while circulating tumor markers are not suitable for early detection, they remain part of the broader spectrum of biomarkers contributing to longitudinal disease assessment in breast cancer.

### 4.5. HER2 Diagnostic Algorithms and ISH Testing

Fluorescence in situ hybridization (FISH) and chromogenic in situ hybridization (CISH) quantify HER2 gene copy number and the HER2/CEP17 ratio. The 2018 ASCO/CAP HER2 testing guidelines introduced five ISH groups based on combined IHC/ISH results, resolving ambiguous cases and defining criteria for HER2 positivity [75]. Dual-probe ISH assays are preferred for their ability to correct for chromosome 17 polysomy. Next-generation sequencing-based HER2 copy number assessment from tumor tissue or liquid biopsy is emerging as an alternative approach, particularly for recurrent or metastatic tissue. However, borderline or equivocal cases may still pose diagnostic challenges, requiring multidisciplinary interpretation.

Together, histopathological evaluation and biomarker profiling form the cornerstone of precision oncology in breast cancer, bridging morphological diagnosis with targeted therapeutic decision-making.

## 5. Molecular Diagnostics and Gene Expression Profiling

Molecular diagnostics have fundamentally transformed breast cancer classification, enabling biologically driven risk stratification and personalized therapeutic decision-making.

### 5.1. Intrinsic Molecular Subtypes

Gene expression profiling studies by Perou, Sørlie, and colleagues defined four principal intrinsic molecular subtypes of breast cancer based on unsupervised hierarchical clustering of the “intrinsic gene list”: Luminal A, Luminal B, HER2-enriched, and Basal-like [4,5]. These subtypes exhibit distinct natural histories, prognoses, and therapeutic vulnerabilities that are imperfectly captured by IHC-based surrogates. Luminal A tumors (ER+, low Ki-67, PR-high) carry an excellent prognosis and respond well to endocrine therapy with minimal benefit from adjuvant chemotherapy. Luminal B tumors (ER+, high Ki-67 or HER2-enriched coexpression) are more proliferative with intermediate-to-poor prognosis. HER2-enriched tumors (ER−, HER2+) respond to HER2-targeted agents. Basal-like tumors, largely overlapping with TNBC, are characterized by high proliferation, TP53 mutations, genomic instability, and sensitivity to platinum-based and anthracycline-taxane chemotherapy [4,5]. Despite their clinical relevance, intrinsic subtypes are not routinely assessed in all settings due to cost and technical limitations.

### 5.2. Multi-Gene Expression Assays

Recurrence Score by Oncotype DX (Genomic Health, Redwood City, CA, USA) is a 21-gene RT-PCR assay generating a recurrence score (RS) of 0–100 validated to predict 10-year distant recurrence risk and chemotherapy benefit in early-stage ER+/HER2−/node-negative breast cancer. The landmark TAILORx trial (*n* = 10,273) demonstrated that women with RS 11–25 derived no chemotherapy benefit beyond endocrine therapy (10-year invasive disease-free survival 84.3% vs. 84.1%), establishing endocrine therapy alone as sufficient for this group, representing approximately 70% of eligible patients [76]. The RxPONDER trial extended Oncotype DX utility to node-positive (1–3 positive nodes) postmenopausal women, showing no chemotherapy benefit for RS ≤ 25, whereas premenopausal women in this group did benefit—possibly due to chemotherapy-induced ovarian suppression [77].

MammaPrint (Agendia, Amsterdam, The Netherlands) is a 70-gene microarray assay classifying tumors as Low-Risk or High-Risk. The MINDACT trial (*n* = 6693) demonstrated that approximately 46% of clinically high-risk tumors were genomically low-risk, and these patients could safely undergo chemotherapy (5-year distant metastasis-free survival 94.7%), validating the test’s prognostic independence from clinicopathological factors [78]. Prosigna (NanoString Technologies, Bothell, DC, USA), the PAM50-based assay generating a Risk of Recurrence (ROR) score and intrinsic subtype classification, and EndoPredict (Myriad Genetics, Salt Lake City, UT, USA), an 11-gene assay incorporating tumor size and nodal status into the EPclin score, complete the commercially validated landscape [79,80]. Among available assays, Oncotype DX is primarily used to guide chemotherapy decisions, whereas MammaPrint provides binary risk stratification, and PAM50-based assays additionally offer intrinsic subtype classification.

Selection among available assays in clinical practice is influenced by regional reimbursement, tissue availability (FFPE sections vs. fresh-frozen), turnaround time, and the specific clinical question—recurrence risk quantification, chemotherapy benefit prediction, or extended endocrine therapy guidance. Ongoing trials are evaluating expanded applications of gene expression and related molecular testing in neoadjuvant and metastatic settings [81]. However, differences in study populations, endpoints, and assay methodologies limit direct comparability between tests. A comparative overview of clinically validated multi-gene expression assays, including their gene composition, validation cohorts, clinical indications, and limitations, is provided in Table 3.

### 5.3. Comprehensive Genomic Profiling by Next-Generation Sequencing

Next-generation sequencing (NGS)-based comprehensive genomic profiling (CGP) of solid tumors interrogates hundreds to thousands of cancer-relevant genes for single-nucleotide variants, indels, copy number alterations, and structural rearrangements simultaneously. FDA-approved CGP assays (FoundationOne CDx, MSK-IMPACT, Tempus xT) detect actionable alterations linked to approved therapies or clinical trial eligibility in a significant proportion of advanced breast cancer patients [82].

Key actionable alterations in breast cancer include: PIK3CA gain-of-function mutations (present in ~40% of HR+ tumors), licensing alpelisib plus fulvestrant (SOLAR-1 trial) [83]; BRCA1/2 pathogenic germline or somatic variants (~5% of all breast cancers), licensing olaparib and talazoparib in the germline-mutated HER2-negative setting (OlympiAD, EMBRACA trials) [84,85]; AKT1 mutations, targetable by capivasertib (CAPItello-291 trial) [86]; NTRK1/2/3 gene fusions (rare, <1%), targetable by larotrectinib or entrectinib [87]; and ESR1 mutations conferring endocrine resistance, detectable primarily in ctDNA from patients progressing on aromatase inhibitor therapy [88]. Tumor mutational burden (TMB) and microsatellite instability (MSI), assessed by NGS, predict response to immune checkpoint inhibitors, though high TMB/MSI-H is uncommon in breast cancer (<2–5%) [89]. The integration of genomic profiling into clinical workflows is increasingly enabling precision oncology approaches in advanced breast cancer. 

### 5.4. Germline Genetic Testing

Germline pathogenic variants in BRCA1 and BRCA2 confer lifetime breast cancer risks of 72% and 69%, respectively, and warrant risk-reduction surveillance and prophylactic surgical counseling [90]. Beyond BRCA1/2, moderate- to high-penetrance genes—PALB2, CHEK2, ATM, CDH1, STK11, and PTEN—are now included in multi-gene panel testing, guided by personal and family history. The 2023 NCCN and ASCO guidelines have expanded genetic testing indications to include all women diagnosed with breast cancer at or before age 50, triple-negative breast cancer regardless of the patient’s age, and any HER2-negative metastatic breast cancer patient regardless of family history, given the therapeutic implications of BRCA1/2 testing [91]. Cascade testing at-risk relatives following identification of a pathogenic variant enables preemptive risk management and has been shown to be cost-effective [92].

Collectively, molecular diagnostics and genomic profiling are redefining breast cancer management, shifting clinical decision-making from morphology-based approaches toward biologically driven precision medicine.

## 6. Liquid Biopsy in Breast Cancer

Liquid biopsy represents a rapidly evolving, minimally invasive approach to cancer diagnostics, enabling real-time molecular profiling and dynamic disease monitoring.

### 6.1. Circulating Tumor DNA

ctDNA refers to tumor-derived cell-free DNA fragments shed into the bloodstream through apoptosis, necrosis, or active secretion by tumor cells. Plasma ctDNA is detectable in 50–80% of metastatic breast cancer patients but in only 10–20% of early-stage patients, reflecting the exponential relationship between tumor burden and ctDNA shedding [93]. Ultra-sensitive detection platforms—digital droplet PCR (ddPCR), BEAMing, and error-corrected tagged-amplicon NGS (eTAm-Seq, iDES)—can detect variant allele frequencies as low as 0.01–0.001%, enabling ctDNA monitoring even in minimal residual disease settings [94].

The clinical applications of ctDNA in breast cancer span multiple settings. In the early-stage disease context, detection of ctDNA at landmark timepoints (post-surgery, mid-treatment) identifies patients with molecular residual disease at high risk of clinical recurrence. The c-TRAK TN trial demonstrated that ctDNA positivity after primary treatment for TNBC predicted early relapses and could trigger clinical assessment, opening pathways for intervention trials [95]. The SERENA-6 trial is evaluating ctDNA-guided endocrine therapy escalation in ctDNA-positive early-stage patients. In the metastatic setting, ctDNA profiling for ESR1 mutations upon progression on aromatase inhibitors guides therapy sequencing (endocrine monotherapy compared with elacestrant therapy, a selective estrogen receptor degrader approved based on ctDNA-detected ESR1 mutation status) [96]. ctDNA allele frequency dynamics correlate with radiological response across multiple studies, serving as an early and pharmacodynamically informative biomarker [97]. However, low ctDNA levels in early-stage disease remain a major technical challenge, limiting sensitivity for early detection. The key clinical trials supporting the implementation of genomic assays and molecularly guided therapies in breast cancer are summarized in Table 4.

### 6.2. Circulating Tumor Cells

CTCs, intact malignant epithelial cells shed from primary or metastatic tumors, were the first liquid biopsy analyte to receive FDA clearance with the CellSearch system in 2004 for prognostic monitoring in metastatic breast cancer [11]. A threshold of ≥5 CTCs per 7.5 mL blood is independently associated with shorter progression-free and overall survival in metastatic breast cancer, providing prognostic information additive to standard imaging. CTC enumeration is limited, however, by low sensitivity in early-stage disease and inability to identify actionable genomic alterations from intact cells without sophisticated single-cell downstream analyses.

CTC-based HER2 discordance, whereby CTCs express HER2 positivity in patients with HER2-negative primary tumors, is observed in approximately 30% of metastatic cases and may reflect clonal selection or phenotypic plasticity, with potential implications for HER2-targeted therapy eligibility in the metastatic setting [98]. The DETECT IV and V trials are investigating anti-HER2 therapy in CTC HER2-positive patients with HER2-negative primary tumors, a paradigm of CTC-guided treatment assignment. Additionally, lack of standardization across detection platforms limits comparability between studies.

### 6.3. Exosomes and Other Liquid Biopsy Analytes

Extracellular vesicles (EVs), including exosomes (30–150 nm) and microvesicles (100–1000 nm), are lipid bilayer-enclosed particles secreted by tumor and stromal cells carrying cargo of DNA, RNA, proteins, and lipids reflective of their cell of origin. Tumor-derived exosomes contribute to breast cancer progression, immune evasion, and pre-metastatic niche formation, and their molecular cargo—including mutant DNA, non-coding RNA, and oncoproteins—constitutes an emerging liquid biopsy analyte [99]. Exosomal long non-coding RNAs and microRNAs have been proposed as diagnostic biomarkers in early-stage breast cancer, though clinical validation remains incomplete.

Cell-free RNA (cfRNA), including messenger RNA and non-coding RNA species in plasma, offers cancer detection signal complementary to ctDNA, particularly for highly expressed tumor transcripts. Multi-analyte liquid biopsy platforms combining ctDNA mutations with protein biomarkers and ctDNA methylation patterns (e.g., CancerSEEK, GRAIL Galleri assay) achieve multi-cancer early detection with organ-of-origin prediction from a single blood test, representing the frontier of population-level cancer screening [100]. The STRIVE and NHS-Galleri trials provide large-scale evidence on MCED performance; 2025–2026 results from PATHFINDER 2 showed a >7-fold increase in cancer detection when added to recommended screening, with high specificity (99.6%), PPV~62%, and a majority of detected cancers in early stages or unscreened types. NHS-Galleri topline (February 2026) reported a 4-fold higher CDR and reductions in Stage IV diagnoses (primary Stage III–IV reduction endpoint not met), with full results pending [101,102]. These findings underscore MCED’s potential for earlier detection while highlighting the need for long-term outcome validation. Importantly, these approaches may enable a shift toward earlier-stage diagnosis across multiple cancer types. However, challenges remain, including cost-effectiveness, risk of overdiagnosis, and integration into existing screening programs. An overview of molecular and genomic diagnostic tools is presented in Table 5.

Collectively, liquid biopsy technologies offer a promising complement to traditional tissue-based diagnostics, with the potential to enable earlier detection, dynamic disease monitoring, and more personalized therapeutic strategies.

## 7. Precision Diagnostics and Therapeutic Implications

### 7.1. Hormone Receptor-Positive/HER2-Negative Breast Cancer

HR+/HER2− disease, comprising the largest breast cancer category, is increasingly managed through biomarker-stratified approaches. The integration of cyclin-dependent kinase 4/6 (CDK4/6) inhibitors (palbociclib, ribociclib, abemaciclib) with endocrine therapy has become the standard of care for advanced HR+/HER2− disease, with progression-free survival benefits of 10–16 months relative to endocrine therapy alone. Companion diagnostic testing for CDK4/6 inhibitor therapy does not currently require a specific biomarker test beyond ER positivity; however, ongoing research is identifying genomic predictors of resistance and sensitivity [103]. ESR1 mutations, arising in ~40% of patients after aromatase inhibitor therapy, confer resistance to aromatase inhibitors but are sensitizing to selective estrogen receptor degraders (SERDs), particularly elacestrant, approved based on ctDNA-detected ESR1 mutation status in the EMERALD trial [96].

PIK3CA mutation testing is required prior to alpelisib administration, using FDA-approved companion diagnostics (therascreen PIK3CA RGQ PCR Kit for tissue and FoundationOne Liquid CDx for plasma). The prevalence of PIK3CA mutations in HR+ breast cancer (~40%) and their diverse distribution across multiple exons necessitate comprehensive mutation profiling rather than hotspot-only testing [83]. Tumor-agnostic approvals for pembrolizumab (TMB-high ≥ 10 mut/Mb) and larotrectinib/entrectinib (NTRK fusion) apply to rare HR+ breast cancer cases harboring these alterations. Collectively, these biomarkers enable increasingly personalized treatment selection and dynamic adaptation of therapy in HR+/HER2− breast cancer.

### 7.2. HER2-Positive Breast Cancer

HER2+ breast cancer has been transformed from the highest-risk to one of the most therapeutically responsive subtypes through sequential HER2-targeted therapies. Pertuzumab-trastuzumab-taxane as first-line metastatic therapy and ado-trastuzumab emtansine (T-DM1) in residual disease after neoadjuvant therapy are established standards. The antibody–drug conjugate trastuzumab deruxtecan (T-DXd, DS-8201) demonstrated landmark overall survival benefit in the DESTINY-Breast03 trial versus T-DM1 in previously treated HER2+ metastatic disease and has redefined the treatment landscape [104]. Critically, T-DXd has demonstrated efficacy in HER2-low (IHC 1+ or 2+/ISH-negative) metastatic breast cancer (DESTINY-Breast04 trial), revolutionizing HER2 diagnostic testing standards and expanding the HER2-targetable population from ~15% to approximately 55–60% of metastatic patients [69].

Accurate HER2 diagnostic testing is therefore more consequential than ever, requiring rigorous adherence to updated ASCO/CAP 2018 guidelines and the emerging HER2-ultralow category. Neoadjuvant chemotherapy with dual HER2 blockade (pertuzumab + trastuzumab) is standard in early HER2+ disease, with pathological complete response (pCR) serving as a validated surrogate endpoint and stratifying the post-surgery therapeutic approach [105]. The ADAPT HER2+/HR− and HER2+/HR+ trials are investigating biomarker-driven treatment de-escalation and escalation based on early on-treatment Ki-67 and pCR assessment. This paradigm shift underscores the transition from binary HER2 classification toward a continuous spectrum of HER2 expression with therapeutic relevance.

### 7.3. Triple-Negative Breast Cancer

TNBC, defined by absence of ER, PR, and HER2 expression, is the most molecularly heterogeneous breast cancer subtype, encompassing six transcriptional subtypes (BL1, BL2, M, MSL, IM, LAR) with distinct drug sensitivities [106]. The identification of PD-L1 expression (CPS ≥ 10 by 22C3 antibody) as a companion diagnostic for pembrolizumab plus chemotherapy in PD-L1-positive early high-risk and metastatic TNBC (KEYNOTE-522, KEYNOTE-355 trials) has introduced immunotherapy as a standard component of treatment for this subtype [81]. Atezolizumab plus nab-paclitaxel approval (IMpassion130) for PD-L1-positive metastatic TNBC was subsequently voluntarily withdrawn, and pembrolizumab remains the approved checkpoint inhibitor in TNBC.

Germline BRCA1/2 testing is obligatory in all TNBC patients regardless of the patient’s age, given the 25–30% prevalence of pathogenic variants and the availability of PARP inhibitor therapy. PARP inhibitors (olaparib, talazoparib) improve progression-free survival in germline BRCA1/2-positive HER2-negative metastatic breast cancer [81,82]. The OLYMPIA trial demonstrated that adjuvant olaparib for one-year reduced recurrence risk in germline BRCA1/2-positive early HER2-negative breast cancer with residual disease or high-risk primary features [107]. Sacituzumab govitecan, an antibody–drug conjugate targeting TROP2, demonstrated overall survival benefit in previously treated metastatic TNBC (ASCENT trial) without a required companion diagnostic [108]. The therapeutic landscape of TNBC is rapidly evolving toward biomarker-driven strategies integrating immunotherapy, DNA repair targeting, and antibody–drug conjugates.

### 7.4. Integrative Multi-Omic Approaches

Large-scale multi-omic characterization studies, including The Cancer Genome Atlas (TCGA) and the METABRIC consortium, have generated comprehensive molecular portraits of breast cancer integrating DNA copy number, somatic mutations, transcriptomics, methylation, proteomics, and microRNA profiling [109]. These datasets reveal cross-omic interactions that refine subtype classification and identify novel therapeutic vulnerabilities. The proteomic layer, accessible through reverse phase protein array (RPPA) and mass spectrometry-based approaches, captures post-translational modifications and pathway activation states that are not inferable from genomics or transcriptomics alone. Spatial transcriptomics technologies (Visium, MERFISH, Xenium) are beginning to characterize the tumor microenvironment with cellular resolution, revealing interactions between malignant cells, cancer-associated fibroblasts, immune cells, and the vasculature that governs therapeutic response and immune exclusion [110]. Such integrative approaches are expected to drive the next generation of precision oncology, enabling patient-specific therapeutic strategies based on multidimensional tumor profiling.

## 8. Risk Assessment and Prevention Strategies

### 8.1. Clinical and Genetic Risk Models

Risk stratification for individualized screening recommendations relies on validated probabilistic models incorporating personal history, family history, mammographic density, hormonal factors, and genetic testing results (Table 6). The Tyrer-Cuzick model (IBIS) and BOADICEA (Breast and Ovarian Analysis of Disease Incidence and Carrier Estimation Algorithm) incorporate multi-gene panel testing results alongside clinical risk factors, enabling estimation of lifetime risk with greater precision than older models (Gail, Bethesda, MD, USA; Claus, Princeton, NJ, USA) that considered only BRCA1/2 [111]. Automated mammographic density assessment tools (Volpara, Wellington, FL, USA; Quantra, Santa Clara, CA, USA) provide continuous volumetric density measurements with superior reproducibility compared with the categorical BI-RADS density classification and are increasingly integrated into risk scoring algorithms.

Polygenic risk scores (PRS) derived from genome-wide association studies (GWAS) of common low-penetrance SNPs (313-SNP and expanding panels) explain approximately 18% of familial risk and stratify the population into quintiles with 3-4-fold differences in lifetime risk between the highest and lowest deciles [112]. Population-level polygenic risk stratification, piloted in the PERSPECTIVE I&I and MyPeBS trials, may enable risk-adaptive screening intervals, more frequent screening for high-PRS individuals and less frequent or no screening for low-PRS individuals, maximizing screening benefit while reducing overdiagnosis and false positives [113]. The integration of clinical, genetic, and imaging-derived parameters is enabling increasingly precise, individualized risk prediction and forms the basis for risk-adapted screening strategies.

### 8.2. Breast Density and Supplemental Screening

Mammographic breast density is an independent risk factor for breast cancer (relative risk 4–6-fold for extremely dense vs. fatty breasts), as well as a determinant of screening sensitivity. Density notification legislation (in force in 38 US states and recommended by the FDA’s updated Mammography Quality Standards Act regulations) requires that women with dense breasts be informed of their density and the limitations of mammography [114]. The resulting decision regarding supplemental screening, ultrasound, CEM, or MRI, should incorporate individual risk level, breast density category, patient preferences, and resource availability, with no single supplemental modality universally superior. In high-risk women, contrast-enhanced MRI remains the most sensitive supplemental modality, whereas ultrasound and contrast-enhanced mammography may provide incremental benefit in intermediate-risk populations. Overall, the shift toward risk-adapted screening and prevention strategies represents a key step toward more efficient, personalized breast cancer care at the population level.

## 9. Future Perspectives

The diagnostic future of breast cancer will be shaped by several converging developments. First, the maturation of MCED blood tests incorporating methylation signatures, fragmentomics, protein biomarkers, and ctDNA mutational signals offers the prospect of cancer detection years before clinical presentation, fundamentally shifting the paradigm from symptom- or imaging-driven diagnosis to pre-clinical molecular detection [100]. Longitudinal implementation studies are needed to characterize optimal integration of MCED tests with organ-specific imaging and the clinical management of screen-detected molecular signals without imaging correlates.

Second, artificial intelligence will increasingly operate not as an isolated image analysis tool but as a component of integrated clinical decision support systems ingesting radiological, pathological, genomic, and clinical data streams to provide holistic, patient-specific risk and treatment recommendations. Federated learning approaches, training AI models across institutions without sharing raw patient data, will accelerate dataset scale while preserving privacy [115]. Third, spatial multi-omics technologies will revolutionize understanding of the breast tumor ecosystem, enabling digital pathology platforms to extract not only tumor cell characteristics but also immune contexture, stromal activation, and vascular architecture from standard tissue sections, providing prognostic and predictive information beyond current IHC and genomic panels [116].

Fourth, the expansion of tumor-agnostic biomarkers (TMB, NTRK, RET, FGFR, HER2 [tumor-agnostic]) will increasingly intersect with breast cancer diagnostics, requiring comprehensive genomic profiling as a standard-of-care investigation for advanced disease regardless of prior histological subtype classification. Fifth, patient-reported outcomes, wearable biosensors, and digital health data integration will complement molecular diagnostics in longitudinal breast cancer management, enabling real-world treatment monitoring and early identification of recurrence outside formal imaging surveillance windows. Sixth, therapeutic resistance diagnostics, tracking ESR1 mutations, CDK4/6 resistance mutations (RB1 loss, CCND1 amplification), HER2 pathway alterations, will become increasingly sophisticated with serial liquid biopsy at each progression event, enabling rational therapy sequencing informed by the real-time molecular evolution of the tumor [117]. Future advancements in breast cancer diagnostics are expected to be driven by the integration of artificial intelligence, radiomics, and multi-omics data. Personalized diagnostic approaches combining imaging and molecular profiling may significantly improve early detection and treatment stratification.

## 10. Conclusions

The diagnostic landscape of breast cancer has evolved from a binary (malignant/benign) determination toward a rich, multiparametric characterization encompassing imaging phenotype, histological grade, receptor expression, gene expression subtype, somatic and germline genomic alterations, immune microenvironment, and liquid biopsy-based residual disease assessment. Each layer of information provides incrementally refining insights into prognosis and therapeutic vulnerability, enabling increasingly individualized treatment decisions. Population screening programs are being augmented by risk-adaptive frameworks that tailor imaging modality, frequency, and supplemental testing to individual risk profiles rather than applying uniform age-based policies. Molecular diagnostics have demonstrated clinically validated ability to spare low-risk patients’ unnecessary chemotherapy, guide targeted therapy selection across all breast cancer subtypes, and identify high-risk patients who benefit from treatment intensification.

Artificial intelligence is poised to democratize expert-level radiological interpretation, improve workflow efficiency, and extract novel imaging biomarkers. Liquid biopsy technologies are transitioning from research tools to clinical instruments for treatment monitoring, early recurrence detection, and companion diagnostic applications. The integration of these diverse diagnostic modalities within electronic health record-linked clinical decision support systems and multidisciplinary tumor board frameworks will be essential to realizing the promise of precision breast oncology in diverse clinical settings. Equitable access to advanced diagnostics across healthcare systems and population groups represents both the greatest challenge and the most consequential imperative for the next decade of breast cancer diagnostic research and implementation. A multimodal diagnostic approach integrating imaging, molecular profiling, and AI-based tools represents the most promising strategy for improving breast cancer detection and personalized patient management.

## Figures and Tables

**Table 1:** Imaging modalities in breast cancer diagnostics.

Modality	Principle	Sensitivity	Specificity	Clinical Indications	Advantages	Limitations
MMG	X-ray imaging	~77–95%	~94–97%	Population screening	Widely available	Reduced sensitivity in dense breasts
DBT	3D MMG	>95%	~94–97%	Screening; dense breasts	Improved lesion detection	Increased radiation dose
USG	Soundwave imaging	~60–95%	~60–90%	Adjunct to MMG	Useful in dense tissue; no radiation	Operator-dependent
MRI	Contrast-enhanced magnetic imaging	>90%	~72–90%	High-risk screening; staging	Highest sensitivity	Cost; false positives
CEM	Iodinated contrast-enhanced X-ray	>90%	>95%	Diagnostic workup	Improved lesion characterization	Contrast exposure

MMG—Mammography; DBT—Digital Breast Tomosynthesis; USG—Ultrasonography; MRI—Magnetic Resonance Imaging; CEM—Contrast-Enhanced Mammography.

**Table 2:** Tissue-based diagnostic biomarkers.

Marker	Method	Clinical Role	Therapeutic Implication
ER	IHC	Hormone receptor status	Endocrine therapy eligibility
PR	IHC	Prognostic marker	Predicts endocrine response
HER2	IHC/FISH	Growth factor receptor	Anti-HER2 therapy
Ki-67	IHC	Proliferation index	Risk stratification
PD-L1	IHC	Immune checkpoint status	Immunotherapy eligibility

**Table 3:** Clinically validated multi-gene expression assays in breast cancer.

Assay	Gene Panel	Key Studies	Clinical Role	Patient Population	Key Findings	Limitations
Oncotype DX (21-gene)	RT-qPCR	TAILORx (*n* = 10,273) [76]; RxPONDER (*n* = 5083) [77]	Predicts recurrence risk and chemotherapy benefit	ER+/HER2−, node-negative and 1–3 node-positive	No chemotherapy benefit for RS 11–25 in postmenopausal patients; predictive of chemo benefit in premenopausal women	Limited utility in HER2+ and TNBC; cost; intermediate-risk interpretation challenges
MammaPrint (70-gene)	Microarray	MINDACT (*n* = 6693) [78]	Binary risk stratification	Early-stage breast cancer (all subtypes, mainly HR+)	Identifies clinically high-risk but genomically low-risk patients who can safely omit chemotherapy	Less predictive of chemotherapy benefit; binary output limits nuance
Prosigna (PAM50)	NanoString nCounter	TransATAC (*n* ≈ 1000) [79]; ABCSG-8 [80]	ROR score	Postmenopausal HR+/HER2− early breast cancer	Provides subtype classification and long-term recurrence risk	Requires specialized platform; less widely used globally
EndoPredict (EPclin)	RT-qPCR	ABCSG-6/8 (*n* ≈ 1700) [80]	Predicts late recurrence risk	ER+/HER2− early breast cancer	Integrates molecular data with tumor size and nodal status for improved prognostic accuracy	Limited predictive value for chemotherapy benefit
Breast Cancer Index (BCI)	RT-qPCR	TransATAC [79]; MA.17 trial [81]	Predicts late recurrence and benefit from extended endocrine therapy	HR+ early breast cancer	Identifies patients benefiting from extended endocrine therapy beyond 5 years	Limited role in chemotherapy decision-making; narrower clinical application

**Table 4:** Key clinical trials supporting molecular diagnostics and genomic-guided therapy in breast cancer.

Trial	Study Population	Sample Size	Study Design	Status	Key Outcomes	Clinical Impact
TAILORx (NCT00310180) [76]	HR+/HER2−, node-negative	10,273	Phase III randomized	Ongoing (not recruiting)	No chemotherapy benefit in RS 11–25 group (postmenopausal)	Established Oncotype DX as standard for guiding chemotherapy decisions
RxPONDER (NCT01272037) [77]	HR+/HER2−, 1–3 positive nodes	5083	Phase III randomized	Ongoing (not recruiting)	No chemo benefit in postmenopausal women (RS ≤ 25); benefit in premenopausal	Extended Oncotype DX use to node-positive disease
MINDACT (NCT00433589) [78]	Early-stage breast cancer	6693	Phase III randomized	Completed	Genomically low-risk patients safely omitted chemotherapy	Validated MammaPrint for risk stratification
SOLAR-1 (NCT02437318) [84]	HR+/HER2− metastatic (PIK3CA-mutant)	572	Phase III randomized	Completed	Alpelisib + fulvestrant improved PFS	Established PIK3CA as actionable biomarker
OlympiAD (NCT02000622) [84]	HER2− metastatic, germline BRCA1/2	302	Phase III randomized	Completed	Olaparib improved PFS vs. chemotherapy	Validated BRCA testing for PARP inhibitor therapy
EMBRACA (NCT01945775) [85]	HER2− metastatic, germline BRCA1/2	431	Phase III randomized	Completed	Talazoparib improved PFS	Confirmed PARP inhibitor benefit in BRCA-mutant disease
CAPItello-291 (NCT04305496) [86]	HR+/HER2− advanced (AKT pathway altered)	708	Phase III randomized	Completed	Capivasertib + fulvestrant improved PFS	Supports AKT1 mutation as therapeutic target
c-TRAK TN (NCT03145961) [95]	Early-stage TNBC (ctDNA-positive)	161	Phase II	Completed	ctDNA positivity predicts relapse; early intervention feasible	Supports ctDNA for MRD detection
SERENA-6 (NCT04964934) [96]	HR+ early-stage (ctDNA ESR1 mutation)	Ongoing	Phase III randomized	Ongoing	Evaluating ctDNA-guided therapy escalation	May establish ctDNA-guided treatment decisions

**Table 5:** Liquid biopsy platforms.

Component	Detection Method	Clinical Role	Current Status
ctDNA	NGS/ddPCR	Mutation detection; monitoring	Emerging clinical use
CTCs	CellSearch, microfluidics	Prognosis; therapy monitoring	Approved in metastatic setting
Exosomal miRNA	RT-qPCR	Early detection	Research stage

**Table 6:** Overview of breast cancer risk assessment strategies and models.

Model/Strategy	Components	Risk Output	Key Advantages	Limitations
Tyrer-Cuzick (IBIS)	Age, family history, reproductive factors, BMI, breast density, genetic testing	10-year and lifetime risk	Incorporates mammographic density and PRS; widely validated	Requires detailed family history; software-dependent
BOADICEA/CanRisk	Family history, genetic variants (including PRS), lifestyle/hormonal factors, density	5-year, 10-year, and lifetime risk	Comprehensive genetic integration: updated versions include PRS and density	Complex; best used with genetic counseling
Gail Model	Age, reproductive history, biopsy history, family history	5-year and lifetime risk	Simple and widely available	Does not include breast density or extended genetics
Polygenic Risk Scores (PRS)	Hundreds of common SNPs from GWAS	Relative and absolute risk strata	Explains ~18% of familial risk; population stratification	Modest discriminative power alone; ancestry bias
AI-Enhanced/Integrated Models	Mammographic features + density + clinical + genetic data	Dynamic/short-term and lifetime risk	Longitudinal analysis; higher accuracy than traditional models	Emerging; needs prospective outcome validation

## Data Availability

No new data were created or analyzed in this study. Data sharing is not applicable to this article.

## Abbreviations

The following abbreviations are used in this manuscript:

AB-MRI	Abbreviated breast magnetic resonance imaging
ABUS	Automated breast ultrasound
AI	Artificial intelligence
ASCO/CAP	American Society of Clinical Oncology/College of American Pathologists
BI-RADS	Breast imaging reporting and data system
BRCA1/2	Breast cancer gene 1/2
CAD	Computer-aided detection
CDK4/6	Cyclin-dependent kinase 4/6
CEM	Contrast-enhanced mammography
cfRNA	Cell-free RNA
CISH	Chromogenic in situ hybridization
CNB	Core needle biopsy
ctDNA	Circulating tumor DNA
CTCs	Circulating tumor cells
DBT	Digital breast tomosynthesis
DCE-MRI	Dynamic contrast-enhanced magnetic resonance imaging
ddPCR	Digital droplet polymerase chain reaction
DENSE	Dense tissue and early breast neoplasm screening
DWI	Diffusion-weighted imaging
ER	Estrogen receptor
ESR1	Estrogen receptor 1
EVs	Extracellular vesicles
FDA	Food and Drug Administration
FFDM	Full-field digital mammography
FISH	Fluorescence in situ hybridization
GLOBOCAN	Global Cancer Observatory
GWAS	Genome-wide association studies
HER2	Human epidermal growth factor receptor 2
HR	Hormone receptor
IHC	Immunohistochemistry
ISH	In situ hybridization
MAM	Mammography
MBI	Molecular breast imaging
MIR	Mortality-to-incidence ratio
MRI	Magnetic resonance imaging
MSI	Microsatellite instability
NCCN	National Comprehensive Cancer Network
NGS	Next-Generation Sequencing
NTRK	Neurotrophic tyrosine receptor kinase
PARP	Poly(ADP-ribose) polymerase
pCR	Pathological complete response
PD-L1	Programmed death-ligand 1
PEM	Positron emission mammography
PI3K	Phosphoinositide 3-kinase
PR	Progesterone receptor
RT-PCR	Reverse transcription polymerase chain reaction
SERDs	Selective estrogen receptor degraders
TCGA	The cancer genome atlas
TILs	Tumor-infiltrating lymphocytes
TNBC	Triple-negative breast cancer
USG	Ultrasound
VAB	Vacuum-assisted biopsy
WHO	World Health Organization
